# Do hypothetical and actual service distances align for urban fringe parks? An investigation of Shanghai using big data

**DOI:** 10.1016/j.heliyon.2024.e37918

**Published:** 2024-09-14

**Authors:** Yi Wan, Siyi Wei, Huantai Harlhax

**Affiliations:** a Department of Landscape Planning and Design, East China University of Science and Technology, Shanghai, 200237, PR China; b The Department of Landscape Study, Tongji University, Shanghai, 200092, PR China

**Keywords:** Urban fringe, Open spaces, Service range, Travel distances, Life circle, Smartphone data

## Abstract

In China's transition from rapid urbanization to high-quality development, urban peripheral parks are increasingly crucial for ecological services and quality of life. However, a disparity persists between their intended function and actual usage, a common occurrence in megacities of China. This study aims to identify the actual service distance and accessibility of parks in fringe areas, using the southwestern sector of Shanghai's main urban fringe as an example. By utilizing mobile location data, it explores the multilevel service attributes of urban fringe parks. Analyzing spatial characteristics from the perspectives of clustering distances and life circle service capabilities, it concludes that: 1) fringe parks exhibit lower service capacities compared to central ones, mainly providing routine services; 2) fixed service range models are inadequate, especially for fringe parks; and 3) community and district-level parks have comparable service distances, highlighting the need to enhance district-level parks. This research aims to optimize park site selection and functions in urban peripheries, providing insights for sustainable planning.

## Highlights

• Urban fringe parks show service range differences compared to central parks.
• Park size affects visitor numbers but not the specific life circle served.
• Urban parks serve dual functions for distant life circles and local residents.

## 1 Introduction

### 1.1 Urbanization and peri-urban parks

As China's urbanization transitions from rapid development to a stage characterized by high-quality advancement [1], the fundamental goals of urban planning and construction are shifting towards high-quality services and enhanced living standards [2]. Urban peripheral areas, serving as transition zones between built environments and natural ecosystems [3], play a vital role in providing urban ecological services [4,5]. Especially in megacities of China, where the scale of built areas is extensive, these peripheral zones (particularly those around the main city) act as critical connectors between the city center and its outskirts [6].

Parks in these peripheral zones are key in delivering urban ecological services and facilitating high-quality lifestyles. Due to their unique location, these parks possess dual characteristics: they function both as urban ecological green spaces and as public open areas [7]. In terms of service delivery, they exhibit spatial service characteristics distinct from those of parks within the central urban areas [8]. They not only cater to city-wide recreational needs [9] but also address the daily leisure requirements of nearby community residents [10,11].

Following the COVID-19 pandemic, there has been a noticeable increase in demand for parks and other green infrastructure [12,13]. Parks in the peripheral areas of major cities must, therefore, utilize their limited land effectively to maximize service utility. However, there are currently discrepancies between the intended functions and actual usage of these peripheral parks, which has result in low service efficiency in many large cities in China [[14], [15], [16]]. To cognize the service scope of fringe parks and improve the park's service efficiency, this paper analyzes the spatial characteristics of service ranges in existing urban peripheral parks, summarizing their service development stages and patterns. The findings regarding the spatial scale and service capabilities are then used to inform spatial layout and functional positioning, proposing strategic adjustments for optimizing the planning of parks in the urban peripheral zones.

### 1.2 Methods for measuring park service ranges

Research on the capabilities of park service ranges often employs accessibility measurement methods based on hypothetical estimates [17]. Accessibility refers to the relative or absolute ease or difficulty of overcoming various barriers to reach a destination from a source, with comparative indicators including distance, time, and cost [18,19]. Common methodologies such as the nearest distance method [20], buffer zone analysis [21], travel cost method, network analysis [22], two-step floating catchment area method [23], and gravity model typically estimate the service capability and ideal resistance differences based on assumed service radii corresponding to the size of parks [18]. Additionally, some studies have calculated the service range of parks based on the “Green Space Planning Standards” [24,25], which posit that [26] “the service outreach of a park is determined by the size of the park and the distance between the park and its users.” However, such assumptions are based on ideal valuations of a park's service capability. In urban peripheral areas where land use is complex and plot sizes vary, accessibility estimation methods can lead to significant deviations.

In recent years, the application of modern information technology and big data [27,28], such as analyzing mobile location data, has enabled more accurate assessments of the actual use of parks [29,30] in urban peripheral areas [31,32]. According to the theory of environmental behavior [33], which posits that the environment profoundly influences human behavior and vice versa, this interaction can modify or even change the nature and meaning of an environment. Following this reasoning, the spatial service characteristics of a park include both the spatial conditions and functional characteristics inherent to the park, as well as the spatial choices and behavioral characteristics of its users. Therefore, by analyzing the spatial characteristics of user behavior, one can further evaluate the spatial characteristics of the service range of parks in urban peripheral areas. This analysis can provide a basis for optimizing the layout and functional positioning of parks in the future.

### 1.3 Development and type of peri-urban parks

Parks in the urban fringe areas vary in size and have evolved differently over time [34]. Large parks often form part of structural ecological green belts [3], such as the Greater London Green Belt, which initially aimed to prevent disorderly urban expansion in 1944; As the city's development needs evolved, its function shifted towards connecting urban open spaces, with the construction of pocket parks and community parks targeting multi-functional beneficial uses becoming a key planning focus from 2021 [35]. Similarly, the Paris Green Belt was initially aimed at controlling urban boundaries in 1987, establishing a green belt of varying widths around urban built-up area [36]. The Berlin-Brandenburg Green Belt was created to address environmental degradation within the metropolitan area in 1988, forming a “patch group green chain” of eight peripheral regional parks, thereby constituting large parks in the urban fringe area [37].

In China, parks in urban fringe areas began with the construction of structural green belts at the end of the 20th century and have undergone 20 years of development and change [38]. The first batch of green spaces in urban fringe areas transitioned from opposing the increment of urbanization and built-up areas to finely enhancing recreational functions in line with urban renewal [20]. For example, Beijing's first green barrier around the central city and Shanghai's outer green belt, both from the 1990s, were repositioned as rings of parks on the fringes of the main city [39].

Small and medium-sized parks in the fringe areas were mostly planned and constructed within the original streets and towns. Compared to the central city, streets, towns, and blocks in the fringe areas are relatively scattered, with sub-centers, residential communities, and industrial parks distributed in cluster formations [40]. This makes the functional zoning of urban fringe areas more distinct compared to the comprehensive land use of central cities [41]. As a result, parks in these areas tend to be concentrated within residential areas and cluster centers, appearing more fragmented compared to central cities.

### 1.4 Research challenge

Based on the measurement methods for the service range of parks and the development history of parks in urban fringe areas, this paper explores the spatial characteristics of the service range of urban fringe parks, raising the following research problems:

(1) What is the actual service range of various urban fringe area parks? Given the uncertainty of the service hinterland of urban fringe parks and the inability to use traditional assumptions to measure the service radius, it is necessary to determine how to quantitatively identify the actual service range of urban fringe parks.
(2) How to describe the spatial characteristics of the service range of urban fringe parks? That is, upon identifying the service range, how to characterize the spatial features of the park's service range, thereby distinguishing the service capabilities of different parks.
(3) Is there a significant difference in the spatial characteristics of the service range between parks in urban fringe areas and central urban areas? Are the current planning standards' scale classifications for urban fringe parks and their service range provisions reasonable? This involves how to reasonably grasp the planning layout and functional positioning of parks.

## 2 Study area and data

### 2.1 Study area

Urban fringe areas possess distinct regional characteristics [42], influenced by the varying urban development backgrounds [43] and stages of different countries and regions [41]. These differences result in unique contradictions, characteristics, causes, and development trends in the fringe areas [44]. In the context of China's socioeconomic development, the urban fringe areas of megacities share some common features: early-stage expansive planning and large-scale expansion; in recent years, with the shift towards stock planning and high-quality construction, the development pace in these areas has slowed [45], with an increased emphasis on ecological construction and the development of parks.

**Fig. 1:**
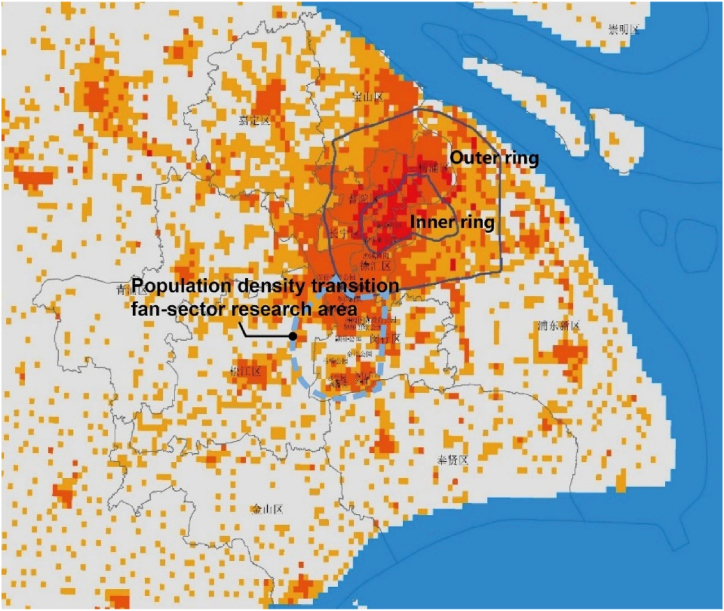
Development form of urban space and scope of inner and outer rings in Shanghai, based on a redrawn map using LandScan 2019 population data.

**Fig. 2:**
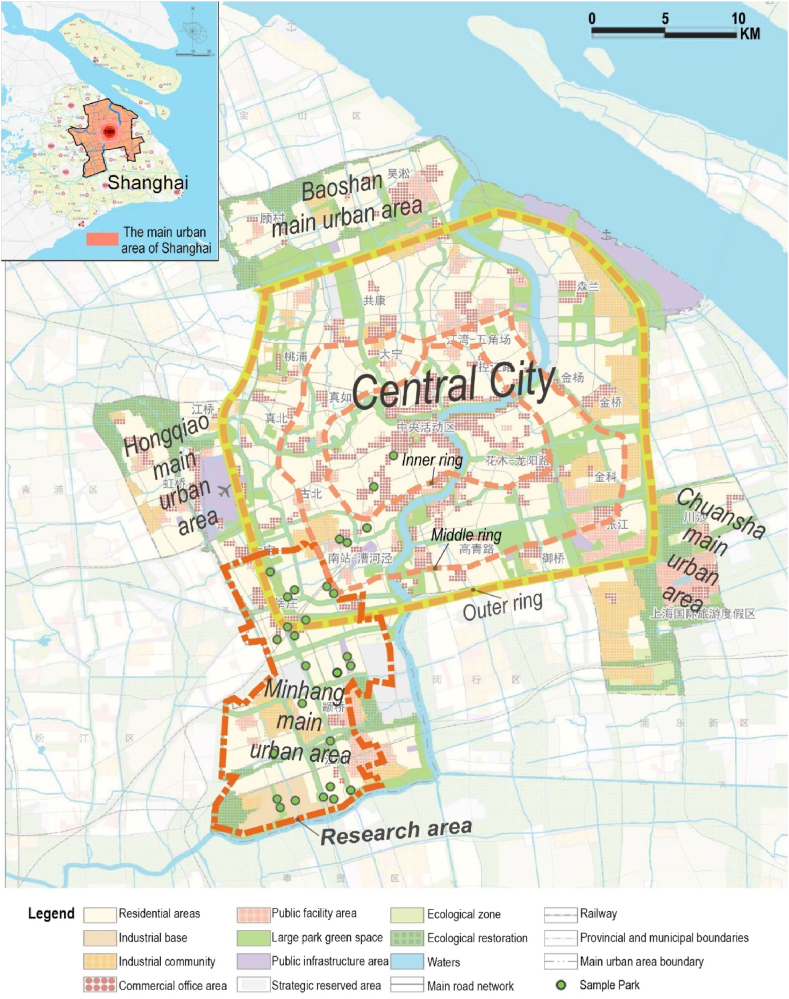
Land layout planning for the main urban area of Shanghai, redrawn according to the master plan of Shanghai (2017–2035).

This paper takes Shanghai as an example, a city with typical characteristics of a megacity's urban fringe of China. According to LandScan population data, Shanghai's urban population is primarily concentrated within the outer ring road, extending westward (Fig. 1). The area near the outer ring road serves as Shanghai's urban fringe transition zone, with the Minhang district in the southwest direction showing continuous development and exhibiting urban-rural transitional characteristics. The Minhang main urban area, a sector of the urban fringe, was selected for study (Fig. 2). Urban fringe areas typically lack clear boundaries, instead exhibiting gradually transitioning characteristics. Thus, the selected sector spans the continuous population concentration at the edge of the city, representing the city's gradual outward transition and its function to provide parks services both inwardly and outwardly. The parks within the study area, being located in the fringe, serve as occasional recreational spots for residents of the central urban area while also providing daily recreational services to the local residential areas [46].

**Fig. 3:**
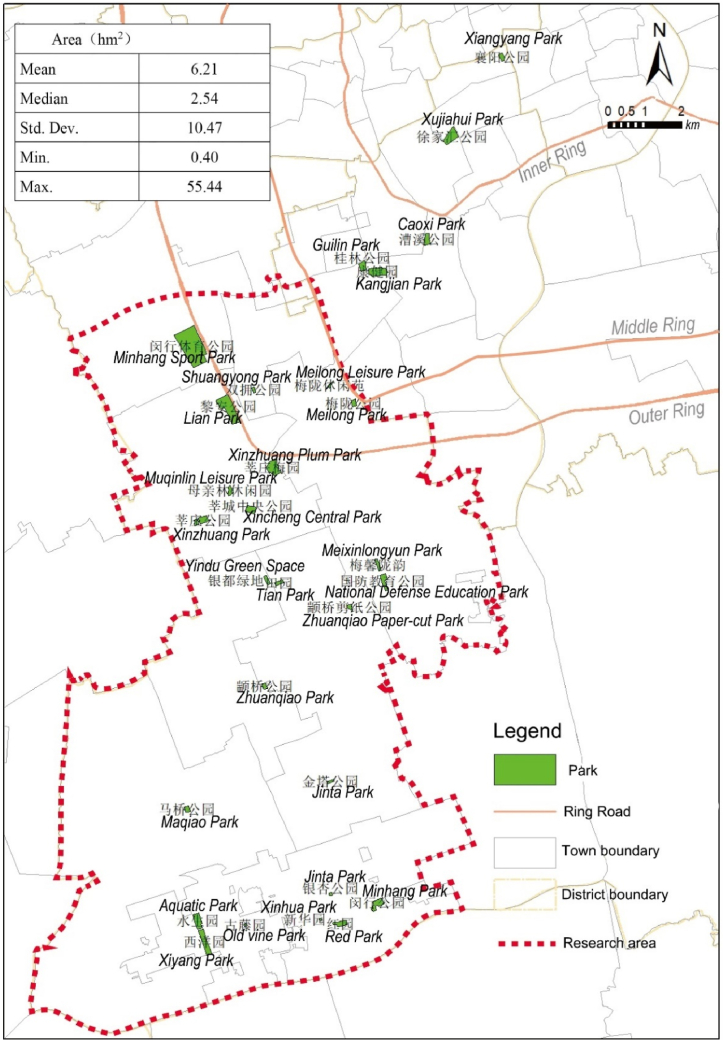
Research scope and spatial distribution of park samples.

For the feasibility and precision of data collection, parks with clearly defined boundaries in the main Minhang district were selected as the study group for urban fringe parks, with a total of park samples. Additionally, 5 parks extending towards the Xuhui district in the central city were selected as a control group within the built-up area (Fig. 3). These selected parks vary in size and level, facilitating a comprehensive and individual analysis of the spatial characteristics of the service range of urban fringe parks [47].

The parks in the study area are categorized according to the urban spatial pattern outlined in the Shanghai Master Plan (2017–2035), ranging from inside the inner ring to between the inner and outer rings, the outer ring green belt, and beyond the outer ring. Beyond the outer ring, based on the planned functional layout, the areas can be subdivided into urban sub-center districts, urban communities, district centers, residential living areas, and industrial communities (Table 1). The scale of the parks, as per the “Shanghai 2035″ master plan's" Parks Classification Standards” (which differ from the “Parks Grading Requirements” in China's “Urban Green Space Planning Standards”), categorizes parks over 50 ha as city parks, those between 4 and 50 ha as district parks, and those from 0.3 to 4 ha as community parks.

**Table 1:**
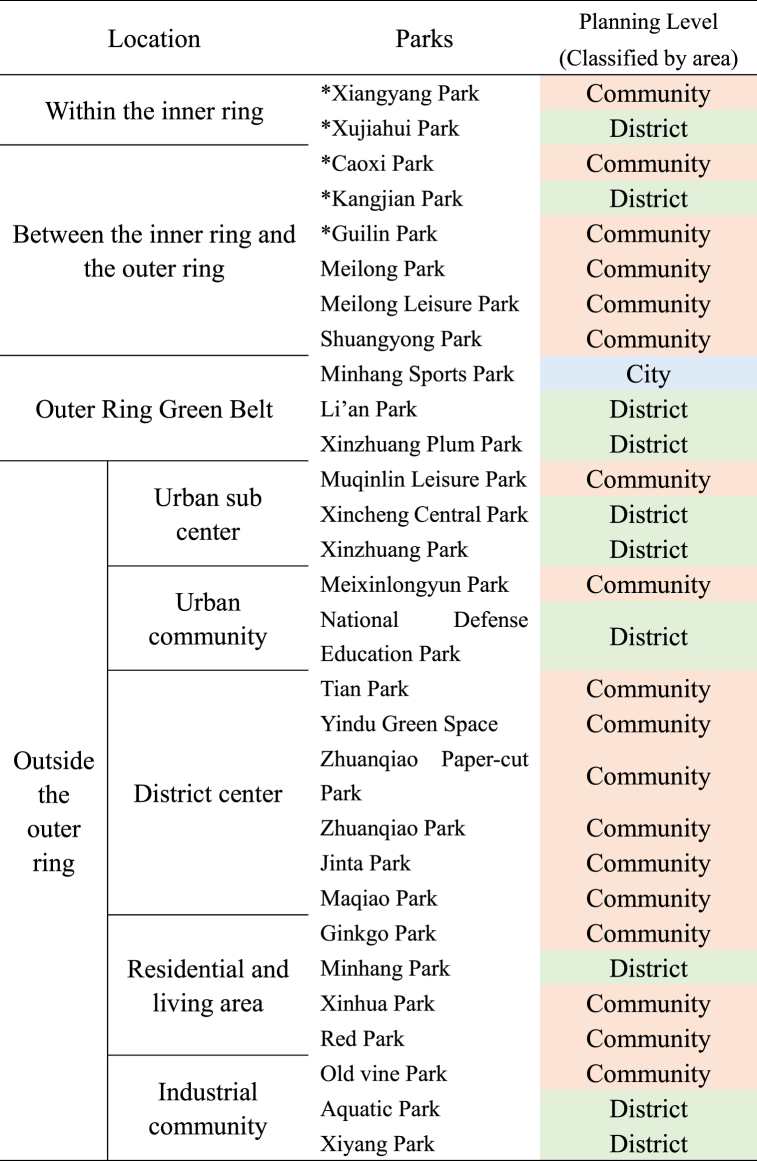
Location and scale grading of parks in research area.

Note: ∗ denotes parks in the central urban area used as a control group.

### 2.2 Data source and processing

The study primarily utilized mobile location data (Location Based Services, LBS), combined with statistical data, to quantitatively analyze the spatial characteristics of the service range of parks in urban fringe areas. The data used in this study includes:

(1) Park Visit Location Data: This refers to the mobile LBS data from October 15 to October 29, 2020, capturing the location data of users who visited 29 selected parks within two weeks. This period was chosen because it represented a stable phase of the pandemic in Shanghai, with park visitor trends largely consistent with previous years, thus minimizing the impact on park activities. The database we used supports approximately 7.6 million users in Shanghai, accounting for 30 % of the total mobile phone users in Shanghai. The mobile device location data obtained in this study was sampled from approximately 30 % of all mobile phone users. The data, which has been anonymized and cleaned, avoids major holidays to reduce significant discrepancies in comparisons between city-level parks and district or community-level parks. The sample involved 25,000 users, resulting in 30,000 park visits, with a total data volume of approximately 6.5 million records. The data fields include uuid, home longitude, home latitude, name of park visited, and time of visit.

The Location-Based Services (LBS) data utilized in this study boasts a precision range of 2–30 m. This level of accuracy significantly surpasses that of mobile phone signaling, which relies on signal stations and offers an average accuracy of 200 m. Such high precision is particularly suited for examining the spatial characteristics of parks and green spaces of varying sizes within this research, especially in studies focusing on the impact radius of smaller parks and green spaces. The data derives from mobile LBS data based on the Android operating system. According to mobile industry data analysis [48], the penetration rate of smartphones in China exceeds 95 %, with Android-based devices constituting 89.6 % of this figure as of 2021. The Android operating system represents the most widely used mobile system among users, achieving a high coverage rate across all age groups. In contrast, iOS, ranking second and exclusive to the iPhone, exhibits a coverage gap and limitations in population reach. Furthermore, a validation comparison between the visitor volume of parks and green spaces derived from the LBS data within this study and related statistical data reveals a consistent trend in data variation, with the location information and current land use characteristics also aligning closely. Therefore, the LBS data adopted in this research ensures its effective value for scholarly investigation.

(2) Visitor Residential Location Data: Derived from mobile LBS point data, this information identifies the residential locations of users who visited parks. The selection criteria for the data include users who visited parks and whose coordinates appeared at the same location for more than 50 days within a three-month period (90 days), specifically between 10 p.m. and 8 a.m. the following day, indicating the location where they spent the longest duration.
(3) Parks Data: Includes the directory and statistical data of parks sourced from the Shanghai Municipal Bureau of Greening and City Appearance. The location and scope data were obtained from sources like Baidu Maps and underwent necessary corrections for accuracy.

## 3 Methodology

### 3.1 Identification methods of park service scope

**Fig. 4:**
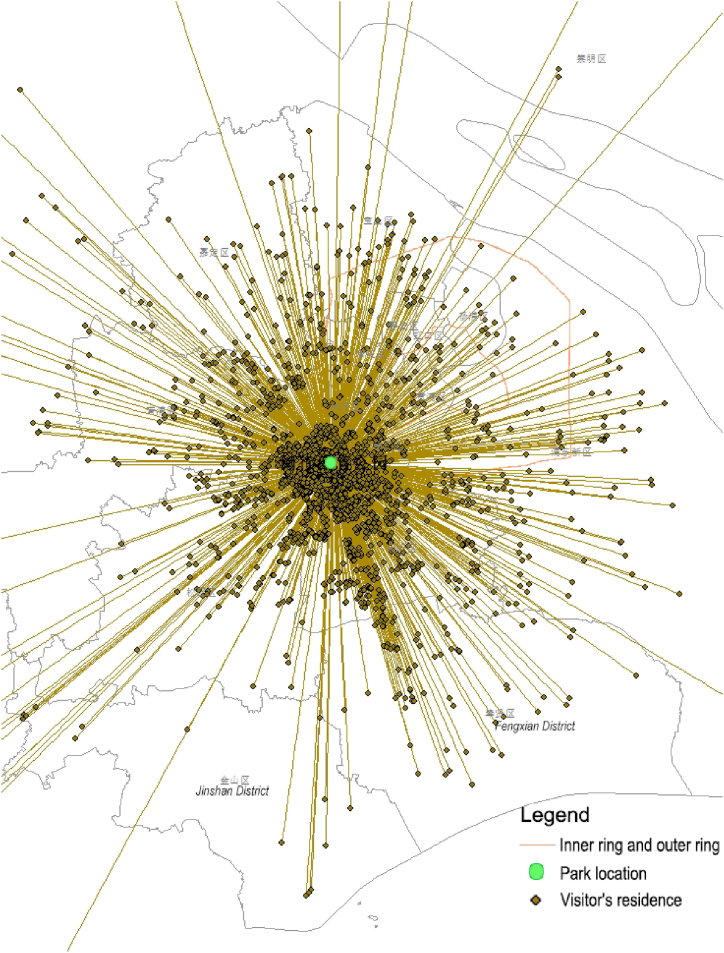
The distance between a visitor's residential location and the visited park exhibits a stratified characteristic.

The method for studying the service range of parks is characterized by the behavioral paths of visitors. By observing the dot distribution maps of the residential locations of the service population for urban fringe area parks, one can note that the service hinterland exhibits characteristics of clustering and stratification (Fig. 4). This suggests that the service range features of parks can be expressed by their service spatial scale and stratification conditions. Additionally, to compare the service ranges of different parks, it is necessary to delve into the deeper characteristics of the service hinterland for spatial measurement. However, the principle of selecting services for 80 % of cumulative visits based on mobile signaling research is not suitable for determining the service range features of parks with significant differences in scale and location. Therefore, linear clustering distance is chosen as the measurement indicator, which, upon validating the spatial characteristic conjectures of park service circle, can also meet the measurement requirements for the multi-scale service hinterland of urban fringe parks. This approach is applicable for benchmarking against existing planning and evaluation standards' accessible radii, thereby facilitating targeted strategy recommendations.

Hence, this paper measures the multi-scale actual service spatial characteristics of parks by using the clustering distance of the residential locations of the service population. Then, based on the threshold values set for different levels of life circles, it assesses the target service range and focus service areas of parks in urban fringe areas.

### 3.2 Clustering distances based on inflection points

By identifying the clustered distribution distances within the service range of parks (i.e., clustering distances), one can determine the proportion of service personnel within different clustering distances, thereby measuring the actual service range characteristics of existing parks.

**Fig. 5:**
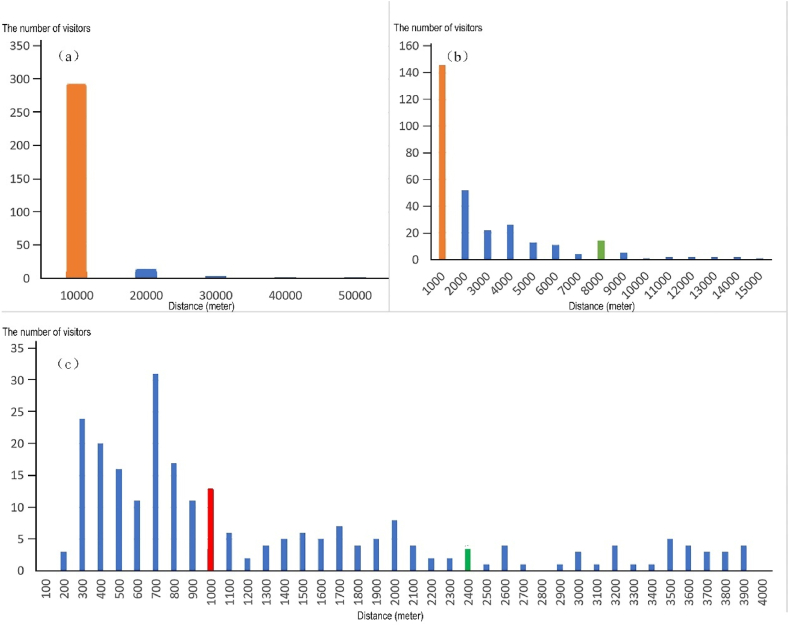
Statistical charts of the number of visitors' residential locations within (a) tens of kilometers; (b) kilometers and (c) hundreds of meters intervals in Xinzhuang Park (Red bars correspond to the 1st clustering distance, green bars to the 2nd and the 3rd clustering distances).

Setting distances in tens of kilometers, kilometers, and hundreds of meters as scale intervals, the number of visitors' residential locations within each distance interval is counted. Through continuous analysis to find inflection points, the cluster distribution distances of actual service populations for each park is identified, revealing the service circle of each park. The principle for identifying inflection points is as follows: if the number of visitors' residential locations drops suddenly after a certain distance interval without a trend of returning to the original level within a short distance, that distance can be determined as the inflection value for clustering circle (Fig. 5). It can be seen from Table 2, the clustering distance (CD) reflects the spatial scale characteristics of the service space of parks. By comparing these with planning objectives, one can evaluate the rationality of park configurations. The actual proportion of people corresponding to clustering circle can illustrate the service influence characteristics of the park service space.

**Table 2:** CD and accumulated proportion of visitors in clustering circles of Xinzhuang park.

Clustering circle	the clustering distance (CD) (m)	The number of visitors	Accumulated proportion
The 1st circle	1000	146	61.34 %
The 2nd circle	2400	210	88.24 %
The 3rd circle	8000	288	90.85 %

### 3.3 Multi life circle assessment of park service ranges

To measure the spatial characteristics of the service ranges of different parks and to standardize comparison [49], this study utilizes the concept of life circles. Each level of life circle corresponds to different needs of the service population [50,51], such as daily activity needs within close community-level life circle, recreational needs at a more distant district-level, and occasional leisure outings at the city-level. Based on previous studies and relevant standards for life circle distances [52,53], the thresholds for the two service range indicators in this study are set at the boundary and suitable values for life circles. The proportion of visitors (PV) in each life circle (city, district, community) corresponds to the number of all visitors' residential points within each life circle range, hence, the maximum boundary value of radius for city, district, and community levels are used as the threshold. The clustering distance (CD) is to measure the clustering attribute of the park service, using suitable radius values for each level (city, district, community) (Table 3). Finally, based on the percentage of population indicators for each life circle level, each park's “key service circle, more important service circle, and general service circle” are defined as belonging to which level of life circle; following the principle that the park's clustering distances exceeds the suitable radius value for the respective life circles, the actual service range corresponding to each park can be determined.

**Table 3:** Threshold values for service range indicators facing life-circle levels.

Life-circle level	Circle radius threshold (boundary value)	Reference value of circle clustering distance (appropriate value)
Community	PV-Com: 0∼900m	CD-1st ≥ 500m
District	PV-Dis: 900m∼4 km	CD-2nd ≥ 2 km
City	PV-Ci: 4 km–20 km	CD-3rd ≥ 10 km

## 4 Results and findings

### 4.1 Clustering distances and service range characteristics

**Fig. 6:**
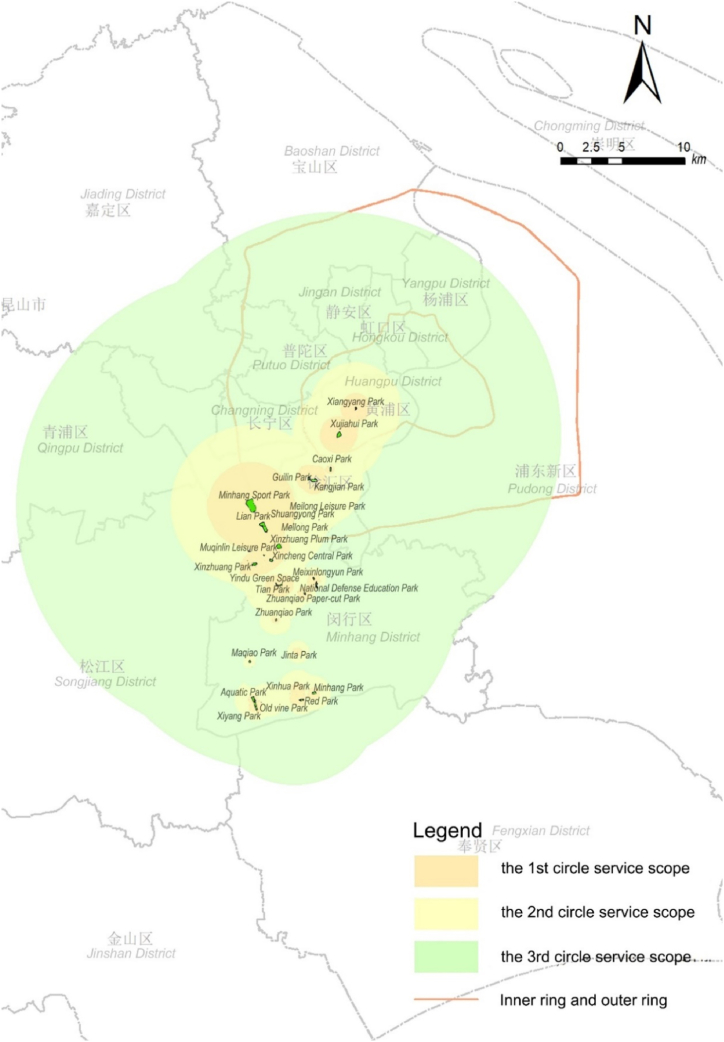

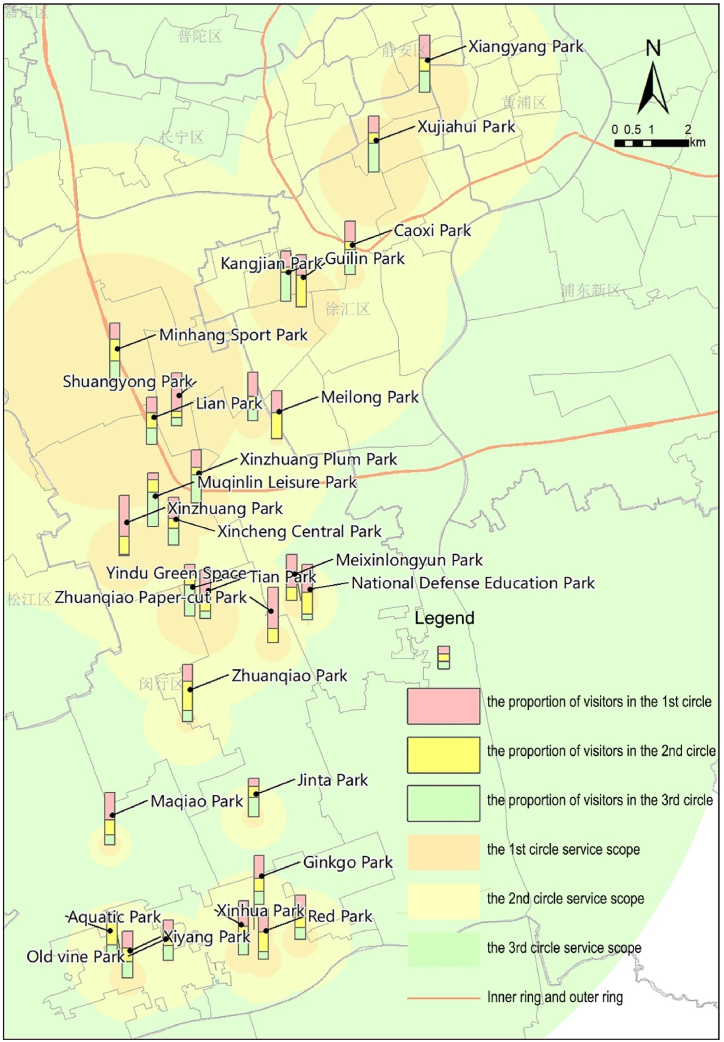
Actual spatial radiation range and population proportion of parks in the southwest sector of Shanghai

Analysis of the clustering distances and their population proportions (Fig. 6) reveals that parks within the study area, although located in the southwest sector of Shanghai, effectively serve an extensive area encompassing the central city and its peripheral southwest regions beyond the outer ring (Fig. 6a). The service ranges of all parks display characteristics of local concentration within the first and second clustering circles, with the third layer extending outward, indicating a broader dispersal.

The proportion of people served in the third layer diminishes as the parks' proximity to the outskirts increases, in contrast to central city parks where the proportion of population in the third layer is significantly higher (Fig. 6b). This indicates that despite the potential for broad service dispersion, the majority of urban fringe area parks serve mainly local residents, with long-distance services being rare occurrences.

### 4.2 Service capabilities for multi-level life circles

**Fig. 7:**
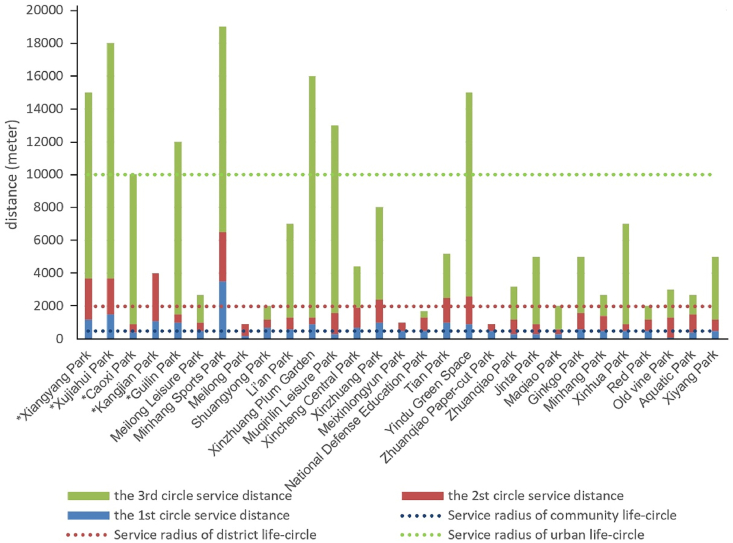
Service radius for life circles and service clustering distances of parks.

Assessing service capabilities of parks against the appropriate radius values for multi-level life circles, we determine that parks exceeding the service radius of a given life circle possess the service capability at that level (Fig. 7), thereby categorizing parks' service capabilities (Table 4). Results show that all central city parks achieve or surpass their planned functions, notably some community-level parks reaching city life circle standards. However, only a few (4/24) urban fringe area parks exceed their planned grade, with more than half (14/24) meeting and several (6/24) failing to reach their intended service level. Thus, service capabilities of parks in urban fringe areas are notably lower than those in the central city, indicating a focus on serving the immediate vicinity and highlighting the need for systemic integration and functional repositioning of some parks.

**Table 4:** Park classification of service capabilities.

Parks	Level of Planning (Classified by area)	Level of Usage (The distance between gathering circles exceeds the corresponding life circle)
aXiangyang Park	Community	City、District↑
aXujiahui Park	District	City、District↑
aCaoxi Park	Community	City↑
aKangjian Park	District	District —
aGuilin Park	Community	City↑
Meilong Leisure Park	Community	Community —
Minhang Sports Park	City	City —
Meilong Park	Community	Community —
Shuangyong Park	Community	Community —
Li'an Park	District	Community↓
Xinzhuang Plum Park	District	Community、City↑
Muqinlin Leisure Park	Community	City↑
Xincheng Central Park	District	Community↓
Xinzhuang Park	District	District —
Meixinlongyun Park	Community	Community —
National Defense Education Park	District	Community↓
Tian Park	Community	District↑
Yindu Green Space	Community	City、District↑
Zhuanqiao Paper-cut Park	Community	Community —
Zhuanqiao Park	Community	Community —
Jinta Park	Community	Community —
Maqiao Park	Community	Community —
Ginkgo Park	Community	Community —
Minhang Park	District	Community↓
Xinhua Park	Community	Community —
Red Park	Community	Community —
Old vine Park	Community	Community —
Aquatic Park	District	Community↓
Xiyang Park	District	Community↓

a denotes parks in the central urban area used for comparison; "↑" and "↓" and indicates that the service capability level is higher and lower than the planned level, respectively; "—" indicates that it is the same as the planned level.

### 4.3 Proportion of visitors and key service life circles

**Fig. 8:**
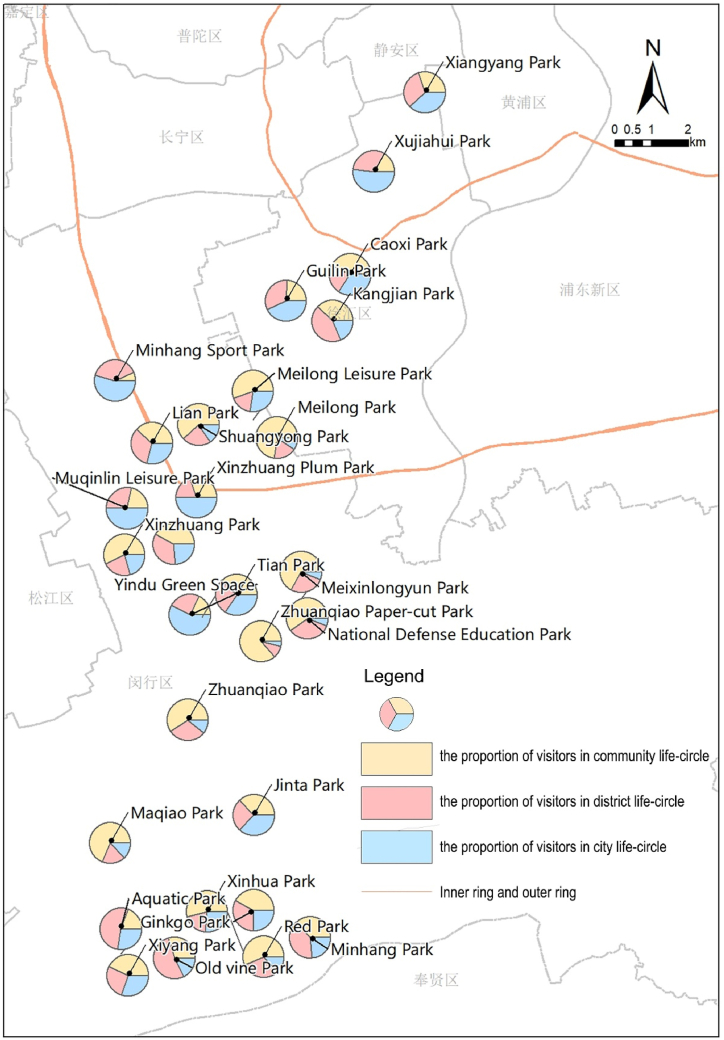
PV within each life circle of parks.

According to the radius thresholds of three life circles, the proportion of visitors (PV) in each life circle is obtained (Fig. 8). Using the natural breakpoint classification method, the proportion of people served by community, district, and city life circles is divided into three levels, representing “non-key service layer, moderately key service layer, and key service layer.” The breakpoints for the community life circle are set at 27 % and 49.4 %, for the district life circle at 22.2 % and 34.4 %, and for the city life circle at 15 % and 29.5 %, identifying parks' key service life circles. Based on these key service life circles, the parks are further categorized into five types based on service range characteristics (Table 5): 1) Diffusion parks extend beyond nearby community life circles, primarily serving distant district or city life circles; 2) Extension parks serve both community and adjacent district life circles, covering a broad service distance; 3) Concentrated parks solely focus on serving nearby community life circles, with a relatively focused service range; 4) Bipolar parks bridge service across both nearby community and distant city life circles, showcasing a dual-distribution of service ranges; 5) Uniform parks evenly serve all three levels of life circles, displaying a Uniform distribution of service range.

**Table 5:** Classification of key service life circles and service scope of parks in urban fringe areas.

Park	Planning Level (Classified by area)	Key service life circle (Key and moderately key services for this life circle)			Characteristic type of service scope
aXiangyang Park	Community	–	District	City	Diffusion
aXujiahui Park	District	–	District	City	Diffusion
aCaoxi Park	Community	Community	–	City	Bipolar
aKangjian Park	District	Community	District	City	Uniform
aGuilin Park	Community	–	District	City	Diffusion
Meilong Leisure Park	Community	Community	–	City	Bipolar
Minhang Sports Park	City	District	–	City	Diffusion
Meilong Park	Community	Community	–	–	Concentrated
Shuangyong Park	Community	Community	–	–	Concentrated
Li'an Park	District	Community	District	City	Uniform
Xinzhuang Plum Park	District	–	–	City	Diffusion
Muqinlin Leisure Park	Community	–	District	City	Diffusion
Xincheng Central Park	District	Community	District	City	Uniform
Xinzhuang Park	District	Community	–	City	Bipolar
Meixinlongyun Park	Community	Community	District	–	Extension
National Defense Education Park	District	Community	District	–	Extension
Tian Park	Community	Community	District	City	Uniform
Yindu Green Space	Community	–	–	City	Diffusion
Zhuanqiao Paper-cut Park	Community	Community	–	–	Concentrated
Zhuanqiao Park	Community	Community	District	–	Extension
Jinta Park	Community	Community	–	City	Bipolar
Maqiao Park	Community	Community	–	–	Concentrated
Ginkgo Park	Community	Community	District	City	Uniform
Minhang Park	District	Community	District	City	Uniform
Xinhua Park	Community	Community	–	City	Bipolar
Red Park	Community	Community	District	–	Extension
Old vine Park	Community	–	District	–	Diffusion
Aquatic Park	District	–	District	City	Diffusion
Xiyang Park	District	Community	District	City	Uniform

a denotes parks in the central urban area used for comparison.

The results indicate that Diffusion and Bipolar parks are predominantly located near district commercial centers and conveniently accessible metro stations, hence more common in the central city. Concentrated and Extension parks are situated within residential areas of urban fringe zones, focusing their services more intensively on the nearby residents. Uniform parks fall into two scenarios: those in the central city, like Kangjian Park, attract a balanced mix of visitors from both near and far, while those in urban fringe areas tend to have fewer visitors, with both near and distant visits being infrequent occurrences. Despite an even distribution of service ratio, the latter scenario may indicate a lower effectiveness in service utility.

## 5 Discussion

This paper addresses the issue of determining the actual service range of parks in urban peripheral areas. It employs quantitative methods to address the uncertainty of service hinterlands and the limitations of using traditional assumptions to calculate service range. Furthermore, based on identifying the service range, the paper describes the spatial service characteristics of parks to distinguish their service capabilities. In describing the spatial characteristics of the service range of urban fringe parks, life circle distance thresholds serve as a standard. This method measures the spatial features of park service ranges, using city, district, and community life circle service distances as benchmarks to classify parks’ service ranges, thus pinpointing the spatial characteristics of park service areas. This study provides valuable insights and guidance for urban planners and landscape architects in urban planning and park site selection.

### 5.1 Significance of life circles for urban fringe parks

We found that parks in urban fringe areas generally exhibit a more concentrated service range compared to those in the city center, with some urban fringe parks failing to meet their planned service range. As shown in Table 6, there is a significant correlation between location and visitor numbers, as well as between location and CD-1st. This indicates that parks farther from the city center have smaller first clustering distance, implying that urban fringe parks have smaller first clustering distances compared to those in the city center. This reflects differences in visitor numbers between central and urban fringe parks, as well as disparities in the range of the first clustering. This aligns with the Central-Periphery Model in urban geography and planning, suggesting active people flow and extended service ranges of parks in the city center, while urban fringe areas, with relatively lower population density, primarily cater to nearby residents. However, this also highlights that the current park system in urban fringe areas remains fragmented and has not fully leveraged its locational advantages to form a cohesive, normative, and interconnected green recreational network.

**Table 6:** Correlations of the service scope indicators for parks.

	Park area	Park location	PV-Com	PV-Di	PV-Ci	CD-1st	CD-2nd	CD-3rd	Visitor volume
Park area	1.00	−0.22	−0.36	0.34	0.30	0.48^b^	0.47^b^	0.32	0.47^a^
Park location		1.00	0.09	0.12	−0.25	−0.47^b^	−0.25	−0.30	−0.59^b^
PV-Com			1.00	−0.41^a^	−0.83^b^	−0.37^a^	−0.67^b^	−0.75^b^	−0.29
PV-Di				1.00	−0.01	0.15	0.56^b^	0.02	0.20
PV-Ci					1.00	0.45^a^	0.51^b^	0.89^b^	0.37^a^
CD-1st						1.00	0.74^b^	0.56^b^	0.53^b^
CD-2nd							1.00	0.58^b^	0.44^a^
CD-3rd								1.00	0.48^b^
Visitor volume									1.00

Spearman's rank correlation coefficient.

a Correlation is significant at the 0.05 level (2-tailed).

b Correlation is significant at the 0.01 level (2-tailed). The indicator of park location is the distance from parks to the city center.

In addition, this suggests that while parks can serve the entire city on an opportunistic basis, normative services are primarily concentrated within specific spatial boundaries. This affirms the significance of serving both close clustering circles and broader life circles. Especially concerning park services in urban fringe areas, the concept of life circles becomes more significant. Previously, there was greater emphasis on life circles within the city center, but as urban development extends outward, inadequate supporting facilities in peripheral areas weaken the life circle. However, the research results indicate a greater concentration of services in peripheral park areas, aligning more closely with the concept of life circles. This contrasts with previous studies that examined how park services in peripheral areas catered to the entire city.

### 5.2 Characteristic relationship between park area and service scope

Parks at the city level, while fulfilling their function of serving distant and higher-level life circles, also play a crucial role in serving residents in their immediate vicinity. Comparing the service radius of life circles with the clustering distances of parks (Fig. 7) reveals that most parks reach the service radius of the community life circle within their first clustering circle, while a smaller proportion (7 out of 29 parks) extending to the community life circle by the second clustering circle. This demonstrates that parks, regardless of their size or location, significantly contribute to serving the nearby community life circle, a characteristic that also applies to parks primarily serving the city life circle. This inherent service to the nearby community life circle remains consistent across parks of varying sizes unless the adjacent residential area and its population seldom utilize these amenities.

Furthermore, it is unreasonable to equate park area with a fixed service range, particularly in the planning of parks located at the urban fringe. Park area is positively correlated with visitor numbers and with the first and second clustering distances. However, its correlation with the proportion of visitors served by the park is not significant. This suggests that park area influences visitor numbers; as visitor numbers increase, parks tend to exhibit characteristics of expanding externalities beyond various levels of service ranges, regardless of the primary life circle served. Similar results have been found in previous studies [41]. Therefore, planning based solely on park size as a criterion for service range is overly absolute.

### 5.3 Re-understand of district parks and future research

**Fig. 9:**
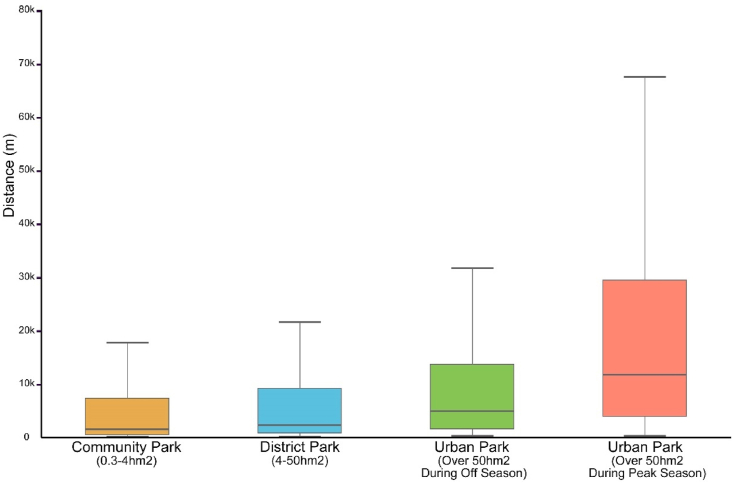
Average median of the actual service range corresponding to the size of parks.

District parks ought to be regarded as venues for routine recreational activities, strategically positioned within a conveniently accessible range of the daily life circle. Through the aggregation of actual distances traversed by park visitors in urban fringe areas and subsequent categorization based on park size, the median distance was ascertained (Fig. 9). The analysis indicates a similarity in distances to both community and district parks, contrasting with distances to city parks, which are two to three times greater. This observation suggests that city parks are perceived as destinations meriting extended travel distances for leisure pursuits, whereas district parks, despite their larger size relative to community parks, continue to serve as venues for everyday recreation. Consequently, it is imperative for district parks to adhere to residential accessibility planning standards, thereby positioning them as spaces intended for routine utilization.

Moreover, the central role of parks in serving a particular life circle is independent of both the size and location of the park, as it is determined by other inherent attributes. These attributes necessitate further research and exploration. In the correlation analysis, the proportion of visitors served by the life circle indicator is not correlated with park size and location. Among them, only the proportion of visitors served by the city life circle is positively correlated with visitor numbers, and simultaneously positively correlated with the clustering distances of the three park services. This indicates that the emphasis of parks on serving the characteristics of the city level is related to visitor numbers, while also exhibiting the characteristics of external expansion of park service clustering distances. It is particularly noteworthy that the proportion of visitors served by the district life circle is only positively correlated with the second clustering distance, but not correlated with the first and third clustering distances, indicating a certain uniqueness of parks serving the district life circle. Compared to the convenient services of the community life circle and the high-level services of the city life circle, the intermediate service characteristics of the district life circle may be related to the environmental conditions within the corresponding range.

(Due to the large amount of data and numerous discrete values, which affect the display of the box plot, the discrete values are not shown in this figure).

## 6 Conclusion

Using the southwestern sector of the main urban area of Shanghai as a case study, this paper quantitatively identifies the actual service range of parks in urban fringe areas based on the multi-level service attributes of parks, leveraging mobile phone location data. Initially, park service ranges at various scales are measured using clustering distances. Subsequently, the service capacity of parks is evaluated using threshold values for community, district, and city-level life circles. Finally, the key service life circles and service range of parks are categorized, and the spatial characteristics of park service ranges are analyzed. The research findings underscore the incomplete nature of the current park system in the urban fringe areas of Shanghai, resulting in relatively low park service utility. Furthermore, the significance of community life circles and broader life circle service levels is affirmed. This study offers specific recommendations for enhancing park accessibility and contributes to the development of effective methods and strategies for implementing the life circle concept, thereby promoting high-quality urban development.

## 7 Limitations

Finally, some limitations of the study must be mentioned. Due to technical reasons for data filtering, this study only focuses on block and point parks, and did not cover the quantification and verification of service coverage for belt green spaces and linear greenways, thereby analyzing and measuring the efficacy and influencing factors of the urban-rural public green space network.

The study uses Shanghai as an example, where the urbanization process is among the most advanced in Chinese cities. The research scope is limited to the urban fringe areas at the edge of the central city, and therefore does not involve rural areas beyond the development boundaries of the urban built-up areas. Future research could consider issues related to rural development under the influence of urbanization, such as the inclusiveness of villages in suburban parks.

Another limitation concerns mobile location data. Although the current usage rate of smartphones in China is over 95 %, there are still a small number of visitors whose samples cannot be obtained through mobile location data. Additionally, the accuracy of the mobile device location data inevitably contains some errors.

## Data availability

The raw data supporting the findings of this study are commercially available from Guangzhou Mengxiang Network Technology Co., Ltd. Due to this, restrictions apply to the availability of the data. The raw data used in this study cannot be shared with third parties. However, the derivative data are available from the authors upon reasonable request.

## Competing financial interests

The authors declare no competing financial interests.

## CRediT authorship contribution statement

**Yi Wan:** Writing – original draft, Methodology. **Siyi Wei:** Investigation. **Huantai Harlhax:** Investigation.

## Declaration of competing interest

The authors declare that they have no known competing financial interests or personal relationships that could have appeared to influence the work reported in this paper.
